# Mapping knowledge structure and emerging trends of extracorporeal membrane oxygenation for acute respiratory distress syndrome: a bibliometric and visualized study

**DOI:** 10.3389/fmed.2024.1365864

**Published:** 2024-07-17

**Authors:** Yanqiu Lu, Wanqing Li, Shaoyan Qi, Kunming Cheng, Haiyang Wu

**Affiliations:** ^1^Department of Intensive Care Unit, The Second Affiliated Hospital of Zhengzhou University, Zhengzhou, Henan, China; ^2^Department of Operating Room, Xiangyang Central Hospital, Affiliated Hospital of Hubei University of Arts and Science, Xiangyang, China; ^3^Department of Orthopaedics, The First Affiliated Hospital of Zhengzhou University, Zhengzhou, China

**Keywords:** acute respiratory distress syndrome, bibliometrics, visualization, hotspots, ECMO

## Abstract

**Introduction:**

With the discovery of extracorporeal membrane oxygenation (ECMO), it is considered as a valuable tool for supporting the treatment of severe acute respiratory distress syndrome (ARDS). It has gained increasing attention, particularly during the COVID-19 epidemic. However, to date, no relevant bibliometric research on the association between ECMO and ARDS (ECMO-ARDS) has been reported. Our study aimed to summarize the knowledge structure and research focus of ECMO-ARDS through a bibliometric analysis.

**Method:**

Publications related to ECMO-ARDS from 2000 to 2022 were obtained from the Web of Science Core Collection (WoSCC). Research data underwent bibliometric and visual analysis by using CiteSpace, VOSviewer, and one online analysis platform. By analyzing the countries, institutions, journals, authors, the geographic distribution of research contributions as well as the leading institutions and researchers in this field were identified. Additionally, prominent journals and highly cited publications were highlighted, indicating their influence and significance in the field. Moreover, the co-citation references and co-occurring keywords provided valuable information on the major research topics, trends, and potential emerging frontiers.

**Results:**

A total of 1,565 publications from 60 countries/regions were retrieved. The annual publication number over time revealed exponential growth trends (R^2^ = 0.9511). The United States was dominant in ECMO-ARDS research, whereas the Univ Toronto was most productive institution. Prof Combes A published the most publications in this area. *ASAIO Journal* and *Intensive Care Medicine* were the most active and co-cited journals, respectively. Reference co-citation analysis showed that current research focus has shifted to COVID-related ARDS, multi-center studies, as well as prone positioning. Apart from the keywords “ECMO” and “ARDS”, other keywords appearing at high frequency in the research field were “COVID-19”, “mechanical ventilation”, “extracorporeal life support”, “respiratory failure”, “veno-venous ECMO”, “SARS-CoV-2”, “outcome”. Among them, keywords like “mortality”, “veno-venous ECMO”, “epidemiology”, “obesity”, “coagulopathy”, “lung ultrasound”, “inhalation injury”, “noninvasive ventilation”, “diagnosis”, “heparin”, “cytokine storm” has received growing interest in current research and also has the potential to continue to become research hotspots in the near future.

**Conclusion:**

This bibliometric analysis offers a comprehensive understanding of the current state of ECMO-ARDS research and can serve as a valuable resource for researchers, policymakers, and stakeholders in exploring future research directions and fostering collaborations in this critical field.

## Introduction

Acute respiratory distress syndrome (ARDS) is an acute diffuse lung injury resulting from various pulmonary or extrapulmonary factors that lead to increased pulmonary vascular permeability, reduced pulmonary compliance and severe ventilation/blood flow ratio imbalance (1–5). Despite advances in clinical understanding and treatment of ARDS, with lung protective ventilation and prone positioning becoming fundamental strategies, ARDS remains a leading cause of mortality in critically ill patients (2). With the discovery of extracorporeal membrane oxygenation (ECMO), it is considered as a valuable tool for supporting the treatment of severe ARDS. It has gained increasing attention, particularly during the COVID-19 epidemic (6, 7). Veno-venous ECMO (VV-ECMO) has been widely employed in the treatment of severe ARDS in recent years, effectively maintaining oxygenation when lung-protective ventilation and conventional treatments prove insufficient (8, 9). V-V ECMO allows to reduce the intensity of ventilation to achieve lung rest while facilitating gas exchange and correcting hypoxemia. Several studies have investigated the benefits of ECMO for patients with respiratory failure. The CESAR multi-center randomized controlled trial demonstrated the potential advantages of ECMO for respiratory failure (10), while the 2018 EOLIA trial supported the beneficial effects of ECMO combined with lung protective ventilation for patients with severe ARDS (11). A meta-analysis comparing VV-ECMO support with conventional mechanical ventilation in ARDS patients revealed lower 60-day mortality rates in the ECMO group (RR = 0·73; *p* = 0·008) (12). With the COVID-19 pandemic, numerous studies have been published in recent years regarding ECMO support in ARDS patients (ECMO-ARDS) (6, 7, 13). A growing number of preclinical and clinical studies are devoted to the field of ECMO-ARDS. However, in the face of massive amounts of information, researchers require extensive time investment to read academic literature to comprehend the latest developments in this field.

Bibliometrics, initially defined by Pritchard (14), is a statistical and mathematical tool for qualitatively and quantitatively evaluating academic literature within a specific field. This analysis enables the identification of trends and hotspots and offers quantitative measurements of research contributions from different countries/regions, journals, institutions, authors, and other relevant details (15, 16). Currently, as a new method, it has been widely applied in critical care medicine (17–21). Take ARDS as an example, multiple researchers have employed bibliometric approaches to investigate the scientific outputs and publication trends of ARDS (17), NLRP3 inflammasome in ARDS (18), ARDS associated with viral pneumonia (19), and so on. However, to our knowledge, no previous bibliometric studies have been conducted to investigate the field of ECMO-ARDS. Therefore, this study employs several bibliometric analysis software, such as CiteSpace and VOSviewer, to analyze and assess literature related to ECMO-ARDS over the past decade. By generating visual graphs, this study aims to explore predominant contributors, current research hotspots, as well as future trends in this field. The main aim of the bibliometric study is to address the following questions:

Q1: How about the global development trend in the field of ECMO-ARDS?
Q2: Who are the main contributors, including authors, institutions, and countries in the field of ECMO-ARDS from 2000 to 2022?
Q3: Which journals are preferred for publishing studies related to ECMO-ARDS?
Q4: What are the main research topics and hotspots in this area?
Q5: What are the most prominent research frontiers and hotspots anticipated in the future?

## Methods

### Data collection

**Figure 1:**
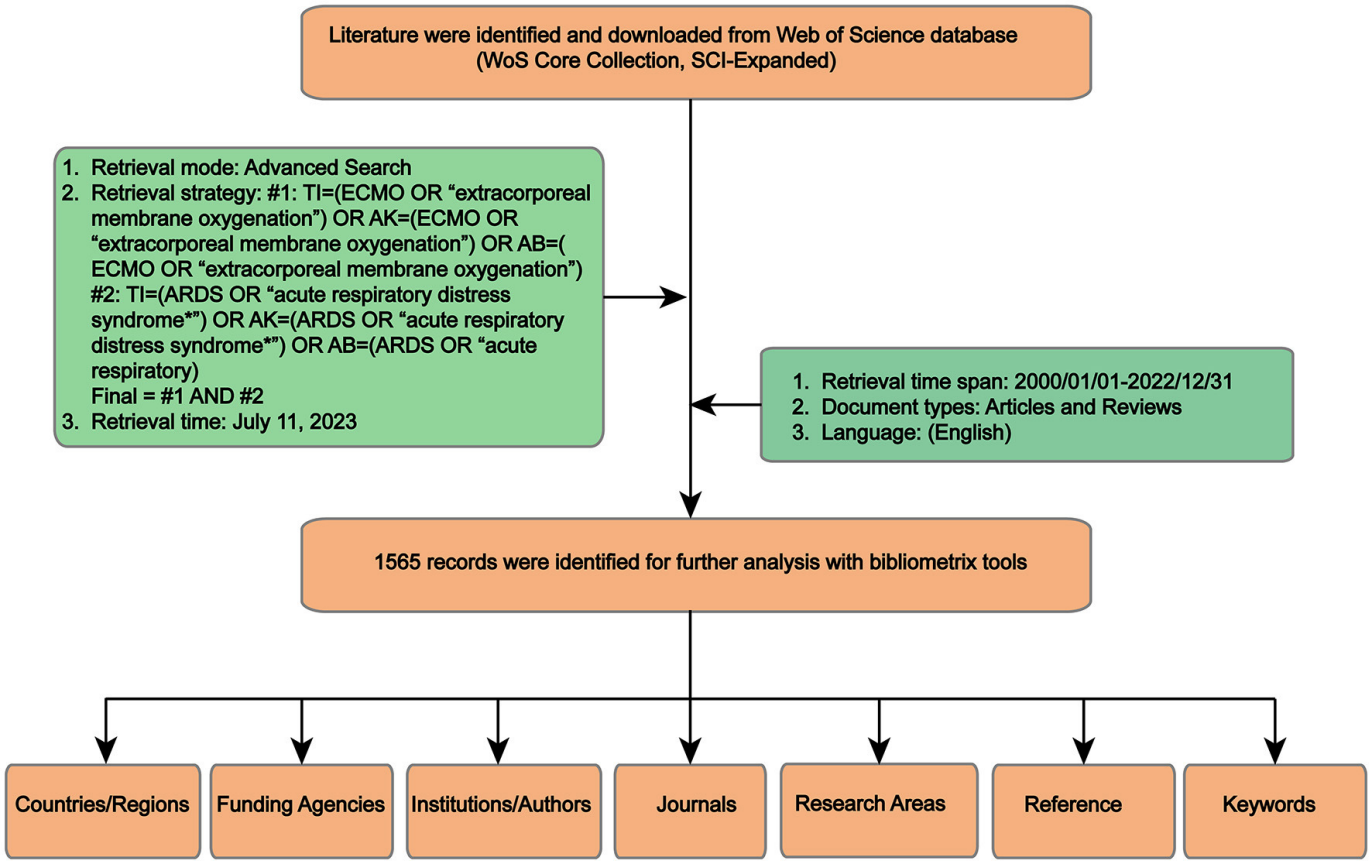
Flowchart diagram of the study.

To minimize potential bias arising from fluctuations in the number of studies and citations, all retrieval operations and data downloads were carried out by two authors within a single day (July 11, 2023). The retrieval strategies and search terms were developed as follows: #1: TI = (ECMO OR “extracorporeal membrane oxygenation”) OR AK = (ECMO OR “extracorporeal membrane oxygenation”) OR AB = (ECMO OR “extracorporeal membrane oxygenation”); #2: TI = (ARDS OR “acute respiratory distress syndrome^*^”) OR AK = (ARDS OR “acute respiratory distress syndrome^*^”) OR AB = (ARDS OR “acute respiratory distress syndrome^*^”); #3: #1 AND #2. A wildcard character (^*^) was utilized to capture variations and variable endings of keywords, allowing for the inclusion of a broader range of data sources. The searched time span was from 2000 to 2022, with language limited to English, whereas the document type was restricted to articles or reviews. All data were downloaded with the record content of “Full Record and Cited References” and exported in tab-delimited format or plain text for further processing using the “Export Records to File” option. Bibliographic related information was extracted and saved in a Microsoft Excel file for further analysis. The study flowchart was shown in Figure 1.

### Data analysis

Bibliometric and visualization analysis were conducted using various software tools including VOSviewer 1.6.16 (Leiden University, the Netherlands), CiteSpace V 6.2 R4 (Drexel University, the USA), and a public visualization platform. Meanwhile, several statistical tools such as SPSS (IBM SPSS Statistics 21, Inc., Chicago, IL, United States) and Microsoft Excel 2019 were also used for descriptive statistical analysis and create graphs. Of them, Microsoft Excel 2019 was used specifically for curve fitting of the annual count of documents and citations. The data was analyzed to find the best-fitting model according to the highest determination coefficient (R^2^). Additionally, the annual average growth rate of papers was calculated using previously described methods (22). This calculation provides insights into the rate at which the number of papers in the field is increasing over the years. The correlation between annual number of publications and citations was assessed using Pearson's correlation coefficient test. A Pearson correlation coefficient value was calculated, and a *p*-value was obtained to determine the significance of the correlation. In this case, correlations with a *p* < 0.05 were considered statistically significant.

The first bibliometric software program developed by van Eck and Waltman (23) is called VOSviewer. It is widely used to visualize and construct bibliometric network maps, including co-authorship, co-citation, and co-occurrence. And offers three types of visualization maps: network, density, and overlay visualization maps, each serving different purposes and conveying different information (24). In these visualization maps, different nodes represent different items such as authors, countries, or institutions, among others. The size of the nodes is determined by the corresponding number of publications, citations, or occurrences associated with the item. The links between nodes represent the relationships or connections between these elements. The relevance between nodes is determined by the total link strength (24). A higher total link strength, suggesting a stronger overall relationship. In this research, we use VOSviewer to conduct paper citation analysis, countries and institutions co-authorship analysis, journals co-citation analysis, as well as co-occurrence analysis of keywords.

CiteSpace software, developed by Chen Chaomei, is a free Java-based bibliometric tool widely used for visualizing and analyzing scientific literature (25). It offers various features for examining the research dynamics and trend in specific topics (26). In this study, CiteSpace was configured with the following settings: time slicing was set from 2000 to 2022, where every 1 or 2 years was regarded as a time slice. The default settings were utilized for text proceedings. In our study, CiteSpace was used to visualize cooperation relationships of institutions and authors, the dual-map overlay of journals, as well as reference and keyword bursts. CiteSpace offers a range of significance metrics that provide insights into different aspects of the analyzed literature. These metrics include both temporal metrics (citation burstness), and structural metrics (betweenness centrality, BC), modularity (the Q score), and silhouette score (the S score) (27–29). BC measures the number of times a node lies on the shortest path between other nodes, based on Freeman's betweenness centrality metric (28). Nodes with high betweenness centrality act as key hubs connecting different clusters within a network. Q score is a metric used to assess the extent to which a network could be divided into distinct modules or clusters. It measures the strength of the division between different groups within the network. The Q score ranges from 0 to +1, with a score closer to +1 indicating a higher level of modularity or clustering within the network (27). A higher Q score suggests a well-structured network with clearly defined clusters. S score is a method for interpreting and validating the consistency within clusters of data. A S score above 0.3 is considered significant, while values above 0.5 or 0.7 indicate reasonable or highly credible clustering, respectively.

In addition, the online bibliometric analytical platform was utilized to analyze collaboration among countries (29). This platform provides specific tools and features to examine and evaluate the collaborative efforts between different countries in the field of interest. Furthermore, an annual publication trend analysis was conducted to assess the publication output and productivity of the countries involved. This analysis allows for the examination of how the number of publications from each country has changed over time, providing insights into their research productivity and contributions in the given field.

## Results

### Global publication outputs and citation trends

**Figure 2:**
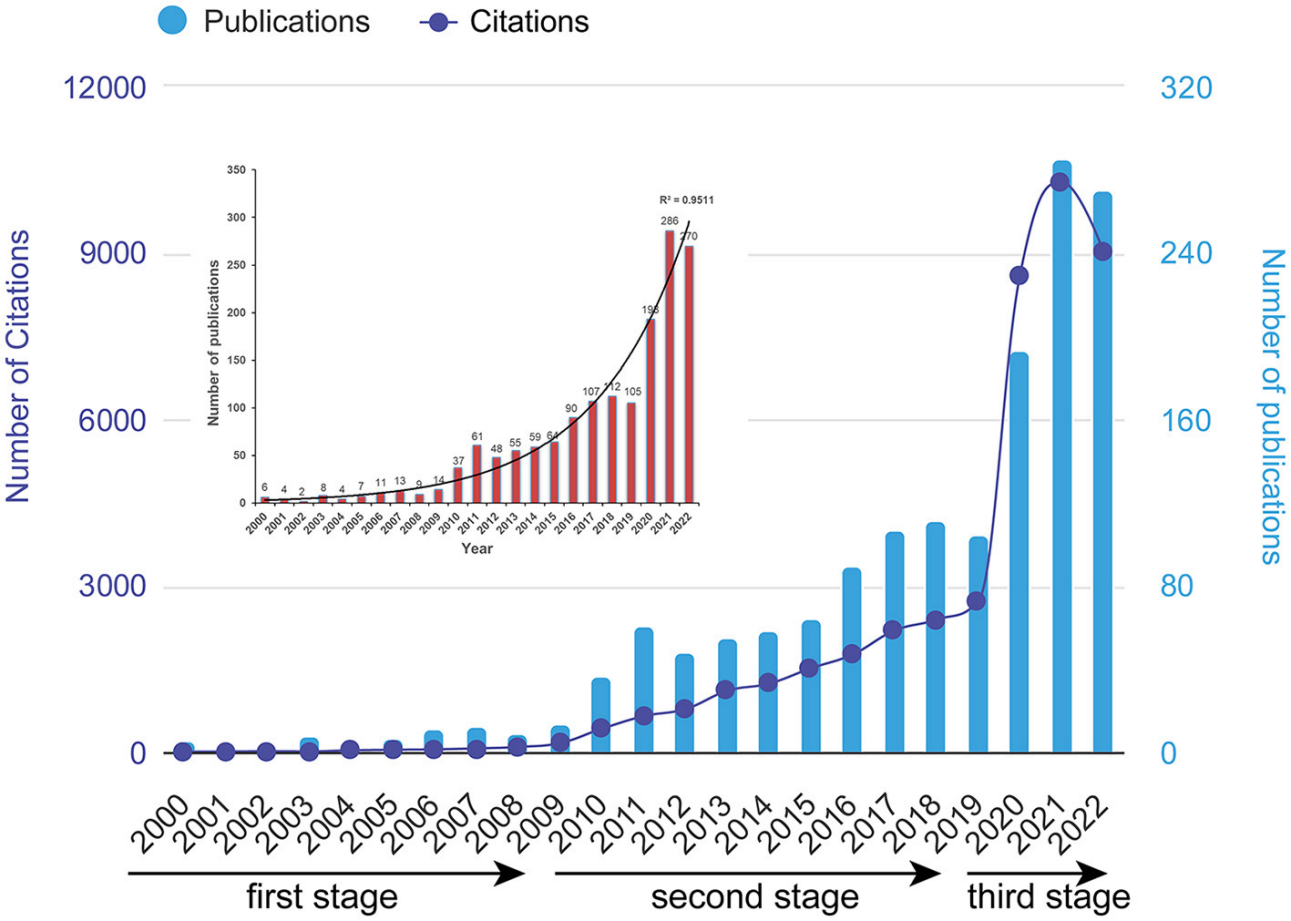
Distribution of annual publication and citation counts of ECMO-ARDS research from 2000 to 2022. The upper left panel represents the trend-fitted curve and correlation coefficients of annual publications.

Based on the literature search and screening strategy outlined in Figure 1, a total of 1,565 documents were finally included. This comprised 1,306 original articles and 259 reviews in the period from 2000 to 2022. The total number of citations for all publications amounted to 46,688 times. On average, each document received approximately 29.83 citations. The H-index of all the selected documents related to ECMO-ARDS was calculated to be 86, indicating that at least 86 articles from the selected publications have been cited at least 86 times each. The publication output increased from 6 in 2000 to 270 in 2022. The average growth rate of publications was calculated to be 18.9%. The trend line analysis conducted on the annual publication number over time revealed exponential growth trends (Figure 2). The determination R^2^ for the annual publication number was found to be 0.9511, indicating a strong fit of the exponential growth model to the data. In addition, we have roughly divided the whole research process of ECMO-ARDS into three periods: first stage (2000–2009), second stage (2010–2019), and third stage (2020–2022). Similarly, the annual number of citations also showed an increasing tendency, aligning with the growth in publication output. Furthermore, the correlation between publications and citations demonstrated a statistically significant correlation (*r* = 0.972, *p* < 0.001).

### Analysis of the most prolific countries/regions and supported funds

**Figure 3:**
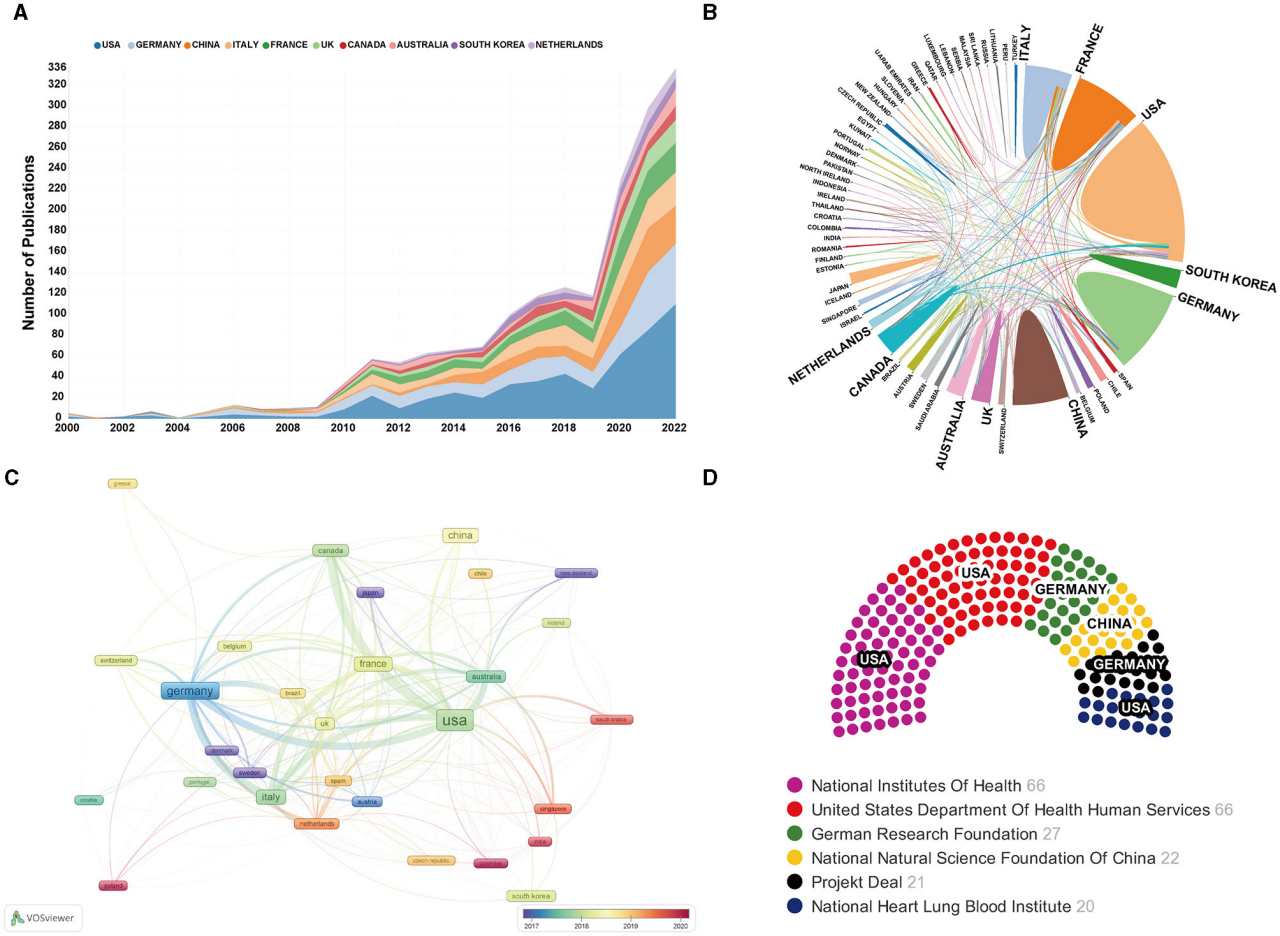
**(A)** Annual publications of the top 10 counties with the most publications from 2000 to 2022; **(B)** Network map of counties/regions collaboration analysis. Lines connecting countries/regions represented their collaborations, and the thickness of the lines represented their collaboration strength. The thicker the line, the closer the collaboration between two counties/regions; **(C)** Overlay visualization map of co-authorship analysis among counties/regions by using VOSviewer. Each node represented a country/region, and the node size is proportional to publication counts. Different nodes are presented by different colors based on the corresponding average appearing year (AAY). Countries/regions assigned with a blue color, signify their early engagement in this field. On the other hand, countries or regions assigned with a red color indicated their relatively later entry. **(D)** The top 6 most active funding agencies.

A total of 60 countries/regions have contributed to papers in this field. Table 1 showed the top 15 contributing countries/regions according to the publication output. The result indicated that the United States is the highest contributor (33.48%) with 524 publications, followed by the Germany (300 articles, 19.17%) and China (192 articles, 12.27%) to the ECMO-ARDS field. Articles from the United States also had the highest H-index (56), and total citations, which was 15,604. Nevertheless, Canada had the greatest average citations per article with 79.75 times, closely followed by France (70.80) and Australia (68.04). Figure 3A listed the annual published studies of the top 10 contributing countries in this field. Figures 3B, C provided visualizations of multinational collaborations between different countries/regions. As can be seen, the most intensive collaborations of the United States were with Canada and Germany. Moreover, in Figure 3C generated by VOSviewer, it color-coded countries/regions according to their average appearing year (AAY). In addition, we also summarized the top 6 related funding agencies in this field (Figure 3D). Remarkably, 3 out of these agencies are headquartered in the United States. Among them, the National Institutes of Health (NIH) tied in first place with the United States Department of Health and Human Services (HHS), both providing support for a significant number of 66 studies.

**Table 1:** Top 15 productive countries in the field of ECMO-ARDS.

**Ranking**	**Countries**	**Publications, *n***	**% of 1565**	**H-index**	**TC**	**AC**
1	USA	524	33.48	56	15,604	29.78
2	Germany	300	19.17	47	7,541	25.14
3	China	192	12.27	27	9,596	49.98
4	Italy	189	12.08	39	5,506	29.13
5	France	164	10.48	47	11,612	70.80
6	UK	102	6.52	26	3,819	37.44
7	Canada	84	5.37	34	6,699	79.75
8	Australia	77	4.92	27	5,239	68.04
9	South Korea	62	3.96	14	574	9.26
10	Netherlands	48	3.07	19	2,205	45.94
11	Japan	45	2.88	12	742	16.49
12	Belgium	42	2.68	17	1,318	31.38
13	Austria	37	2.36	13	914	24.70
14	Spain	31	1.98	16	1,002	32.32
15	Sweden	29	1.85	13	664	22.90

Ranking: TC, Total citations; AC, Average citations per document.

### Analysis of the most productive institutions

**Figure 4:**
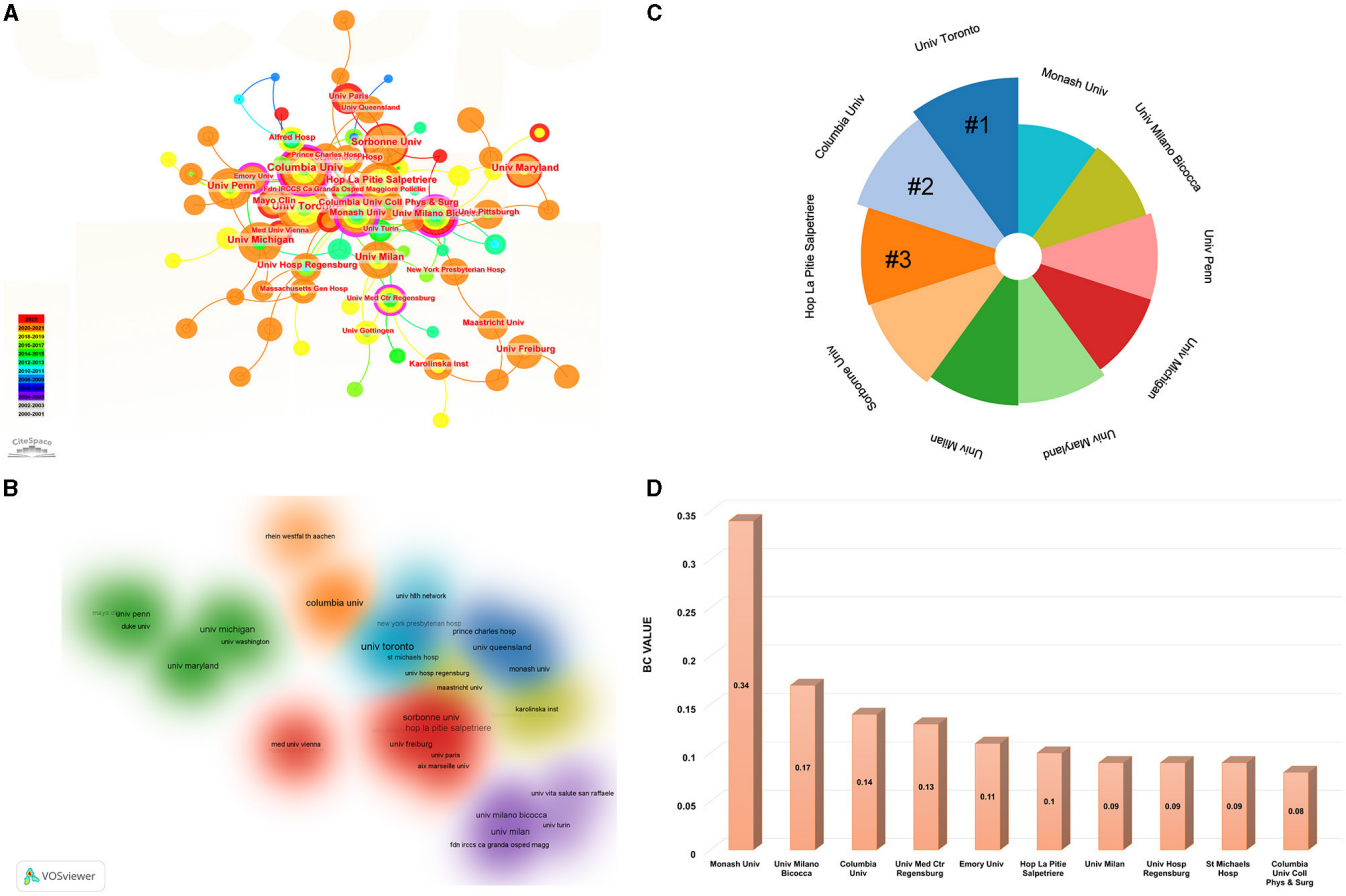
**(A)** Network map of institutional collaboration analysis generated by CiteSpace. The size of each node corresponds to the number of publications, with larger nodes indicating higher publication counts. Nodes with high BC values (BC ≥ 0.1) are displayed with a purple ring. **(B)** Cluster visualization map of institution co-authorship analysis. Institutions with close collaborations were assigned the same color. **(C)** Top 10 institutions with the most publications. **(D)** Top 10 institutions with the highest BC values.

According to our statistical data, over 1,800 institutions have contributed to research in this area. Of them, Figure 4C listed the top 10 institutions with the most publications. The top 3 institutions by publication volume were Univ Toronto, Columbia Univ, and Hop La Pitie Salpetriere. Figures 4A, B provided visualizations of collaborations between different institutions. As shown in Figure 4A, institutions with purple outer rings on the nodes signify high centrality, denoted by a centrality value >0.1. Figure 4D specifically highlighted the top 10 institutions with the highest BC values. Among them, Monash Univ (0.34), Univ Milano Bicocca (0.17), and Columbia Univ (0.14) occupied the top 3 positions. Additionally, the cluster visualization map generated by VOSviewer in Figure 4B is another way to identify clusters of co-operation. As can be seen, a total of 7 research clusters, denoted by the same color, have been identified.

### Analysis of the most influential authors

**Figure 5:**
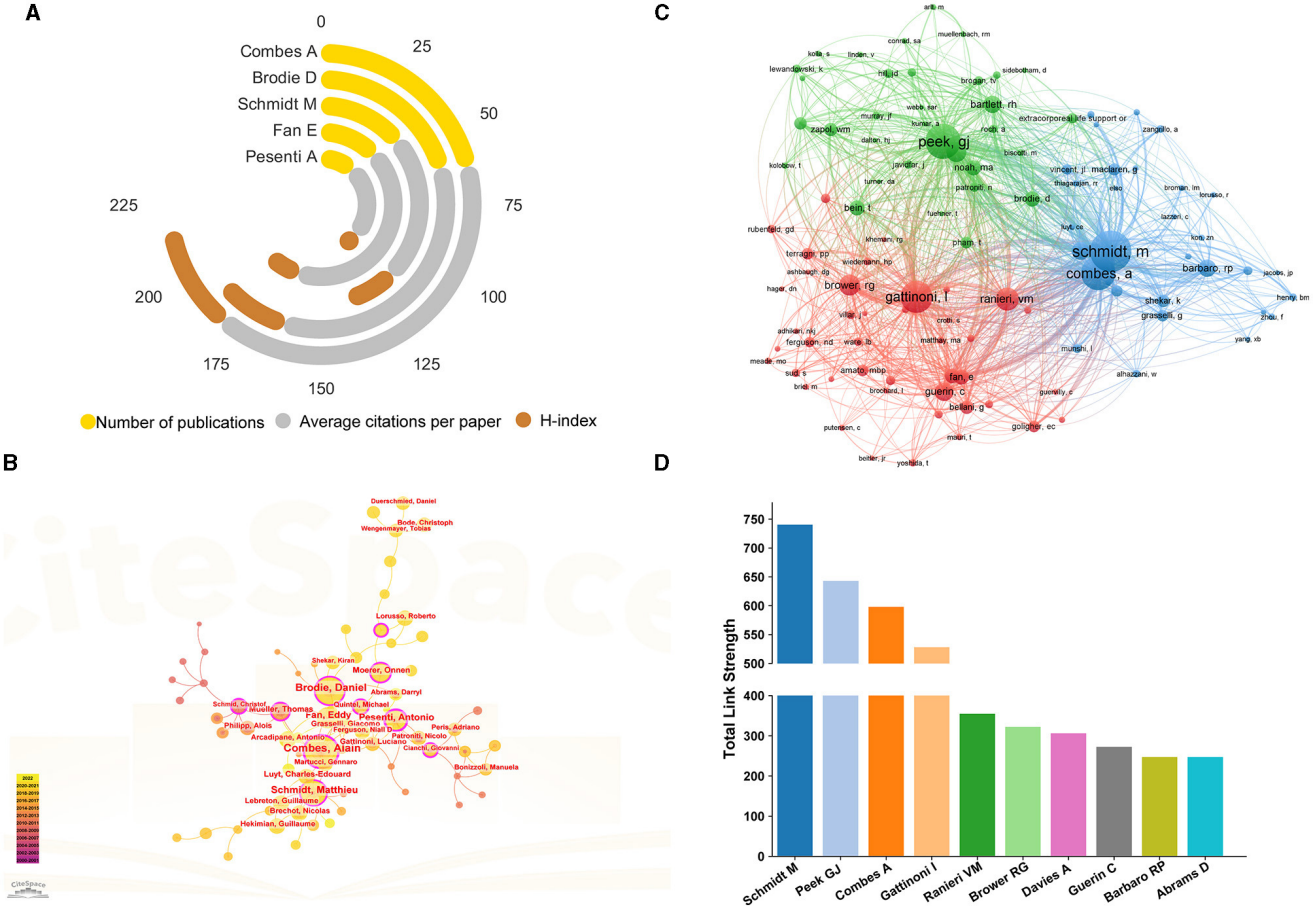
**(A)** Top 5 productive authors and their H-index, average citations per paper. **(B)** Network map of author collaboration analysis generated by CiteSpace. **(C)** Network visualization map of author co-citation analysis generated by VOSviewer. Each node represented an author, and the node size is proportional to citation counts. Distance between two nodes reflects the relatedness of their co-citation link, and nodes with stronger correlation will be assigned to one cluster with the same colors. **(D)** Top 10 authors with the highest total link strength.

The identification of influential authors in specific field is of great importance in scientific research. Influential authors are those who have made significant contributions to their fields and have a substantial impact on the advancement of knowledge and understanding. Figure 5A listed the top five productive authors who published the greatest number of studies and their H-index, average citations per paper. It can be seen that Combes A, Brodie D, and Schmidt M published the most relevant papers, with at least 42 publications. Nevertheless, among them, Fan E from Univ Toronto had the highest mean number of citations with 126.05. Figure 5B demonstrated the network map of author collaboration analysis generated by CiteSpace. Several authors such as Combes A, Pesenti A, Schmidt M, Mueller T, as well as Brodie D, et al., occupied the central positions in the collaboration network, with the BC values in these authors >0.1. Further author co-citation analysis was conducted by VOSviewer, and there were 108 nodes and 3 clusters in this network map (Figure 5C). Authors with more than 50 citations were analyzed and the top 3 authors with the largest Total Link Strength (TLS) were Schmidt M, Peek GJ, and Combes A (Figure 5D).

### Analysis of the higher-impact journals and active research areas

**Figure 6:**
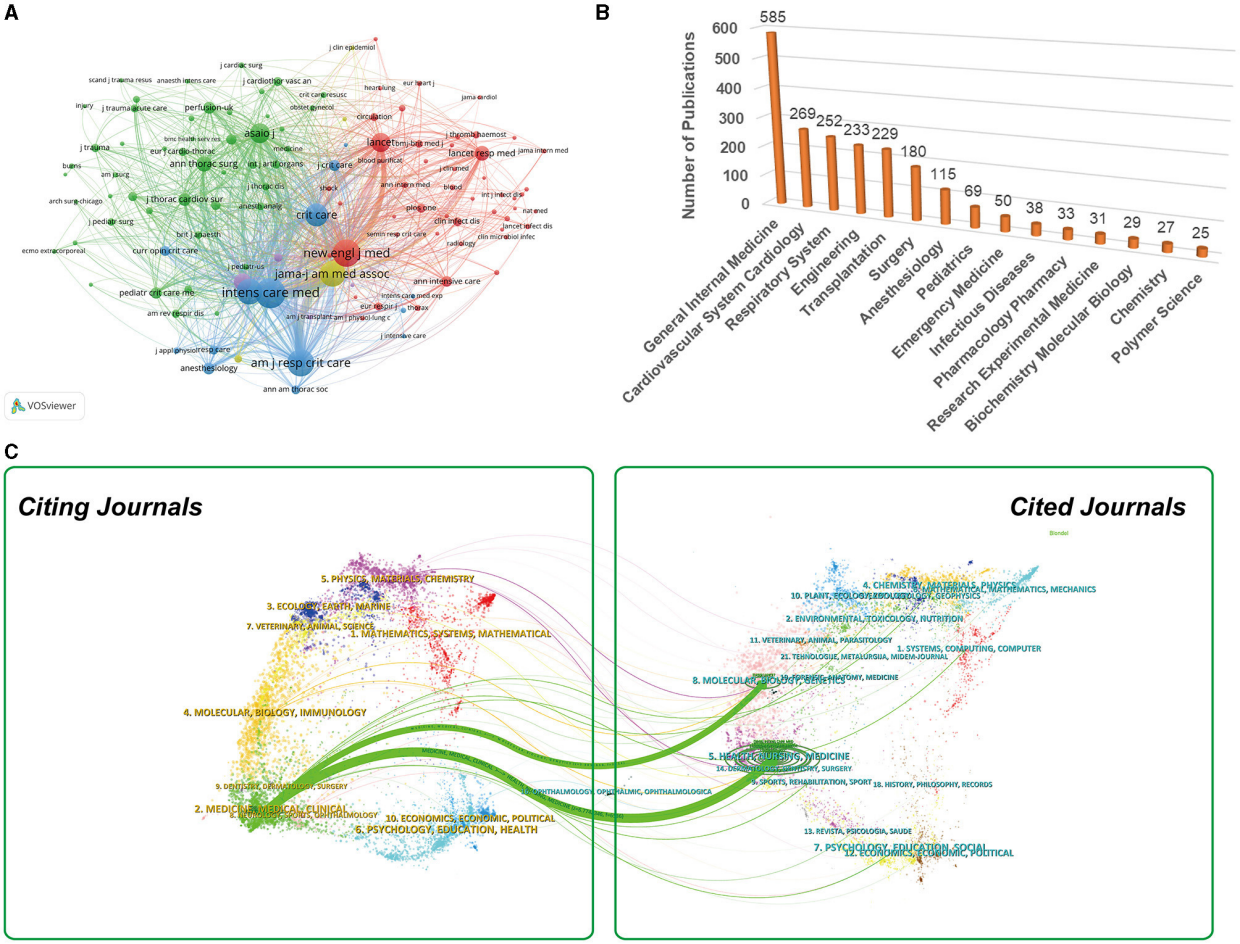
**(A)** Network visualization map of journal co-citation analysis generated by VOSviewer. **(B)** Top 15 active research areas in the field of ECMO-ARDS. **(C)** The dual-map overlay of journals regarding ECMO-ARDS. There are two main citation pathways in this map.

Table 2 provided a comprehensive summary of key information regarding the top 15 most active journals in this field. Notably, *ASAIO Journal* had published the highest number of studies (109), followed by *Perfusion UK* (83), *Critical Care* (54), and *Critical Care Medicine* (53). Of the top 15 scientific journals, *Intensive Care Medicine* (38.9) had the highest IF, followed by *American Journal of Respiratory and Critical Care Medicine* (24.7), and *Critical Care* (15.1). Figure 6A provided network map of the 119 co-cited journals that received more than 50 citation times. Among these co-cited journals, the top three most frequently cited were *Intensive Care Medicine, The New England Journal of Medicine*, and *American Journal of Respiratory and Critical Care Medicine*. Figure 6B provided an illustration of the top 15 research areas of ECMO-ARDS based publication quantity. The research areas that acquired the most focus were General Internal Medicine, Cardiovascular System Cardiology, Respiratory System, Engineering, and Transplantation. Figure 6C illustrated a dual-map overlay depicting the journals involved with ECMO-ARDS. The map revealed 2 primary citation paths within the current network.

**Table 2:** Top 15 most active journals in ECMO-ARDS field.

**Ranking**	**Sources title**	**Output**	**% of 1565**	**IF 2022**	**JCR quartile 2022**
1	*ASAIO Journal*	109	6.96	4.2	Q2/Q2
2	*Perfusion UK*	83	5.30	1.2	Q4/Q4
3	*Critical Care*	54	3.45	15.1	Q1
4	*Critical Care Medicine*	53	3.39	8.8	Q1
5	*Intensive Care Medicine*	43	2.75	38.9	Q1
6	*Artificial Organs*	42	2.68	2.4	Q3/Q3
7	*Journal Of Cardiothoracic And Vascular Anesthesia*	38	2.43	2.8	Q3/Q3/Q3/Q3
8	*Current Opinion In Critical Care*	28	1.79	3.3	Q2
9	*Frontiers In Medicine*	28	1.79	3.9	Q2
10	*International Journal Of Artificial Organs*	28	1.79	1.7	Q4/Q3
11	*Journal Of Clinical Medicine*	28	1.79	3.9	Q2
12	*Journal Of Critical Care*	26	1.66	3.7	Q2
13	*Annals Of Intensive Care*	25	1.60	8.1	Q1
14	*American Journal Of Respiratory And Critical Care Medicine*	24	1.53	24.7	Q1/Q1
15	*Membranes*	24	1.53	4.2	Q2/Q2/Q2/Q2

### Highly cited papers

**Figure 7:**
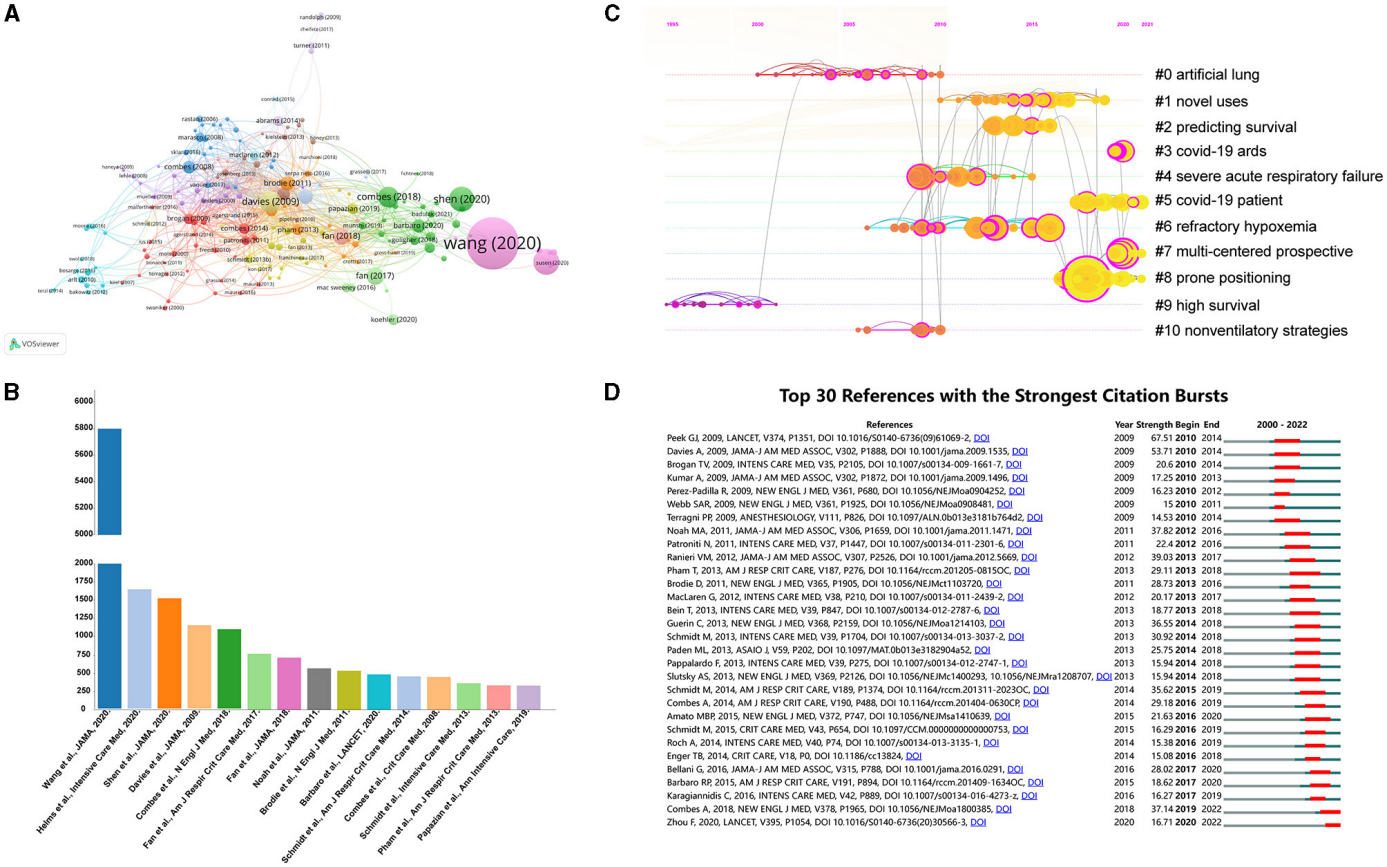
**(A)** Network visualization map for document citation analysis. **(B)** Top 15 highly cited papers in this field. **(C)** Timeline view network map of co-cited references generated by CiteSpace. **(D)** Top 30 references with the strongest citation bursts. The red bars represented the burst period including the beginning and the end year.

Figure 7A illustrated the network visualization map for paper citation analysis. larger nodes typically represent papers with higher citation counts, which indeed signifies the impact and influence of those papers within the citation network. In addition, Figure 7B showed the details of the top 15 highly cited studies in the research scope of ECMO-ARDS. More than half of the articles were published after 2017. Of these, 5 papers were cited more than 1,000 times with all the top 15 were cited over 330 times. The publication authored by Wang et al. (30) stands as the most highly cited, receiving 5,801 citations. In the second position was the study by Helms et al. (31), which garnered 1,651 citations. Following closely behind, in the third position, was the paper authored by Shen et al. (32), with 1,526 citations.

### Co-cited references and references burst analysis

Reference co-citation analysis, a notable function of CiteSpace, is commonly used to identify research focuses in a given field. Figure 7C displayed a reference co-citation network map, comprising 11 major clusters. The mean S value was calculated to be 0.9317, and the mean Q value is 0.7743, indicating the rationality of the clustering strategy. Moreover, this map has provided a timeline view for these clusters. Based on the time axis or the average year of the clusters, this timeline view allows for a quick assessment of the evolution characteristics of each cluster. One can see that the current research focus has shifted to COVID-related ARDS, multi-center studies, as well as prone positioning. In addition, references with citation bursts indicate that certain studies have garnered significant attention from the academic community during specific periods. As presented in Figure 7D, we identified the top 30 references with the strongest citation bursts.

### Keywords co-occurrence analysis and burst keywords

**Figure 8:**
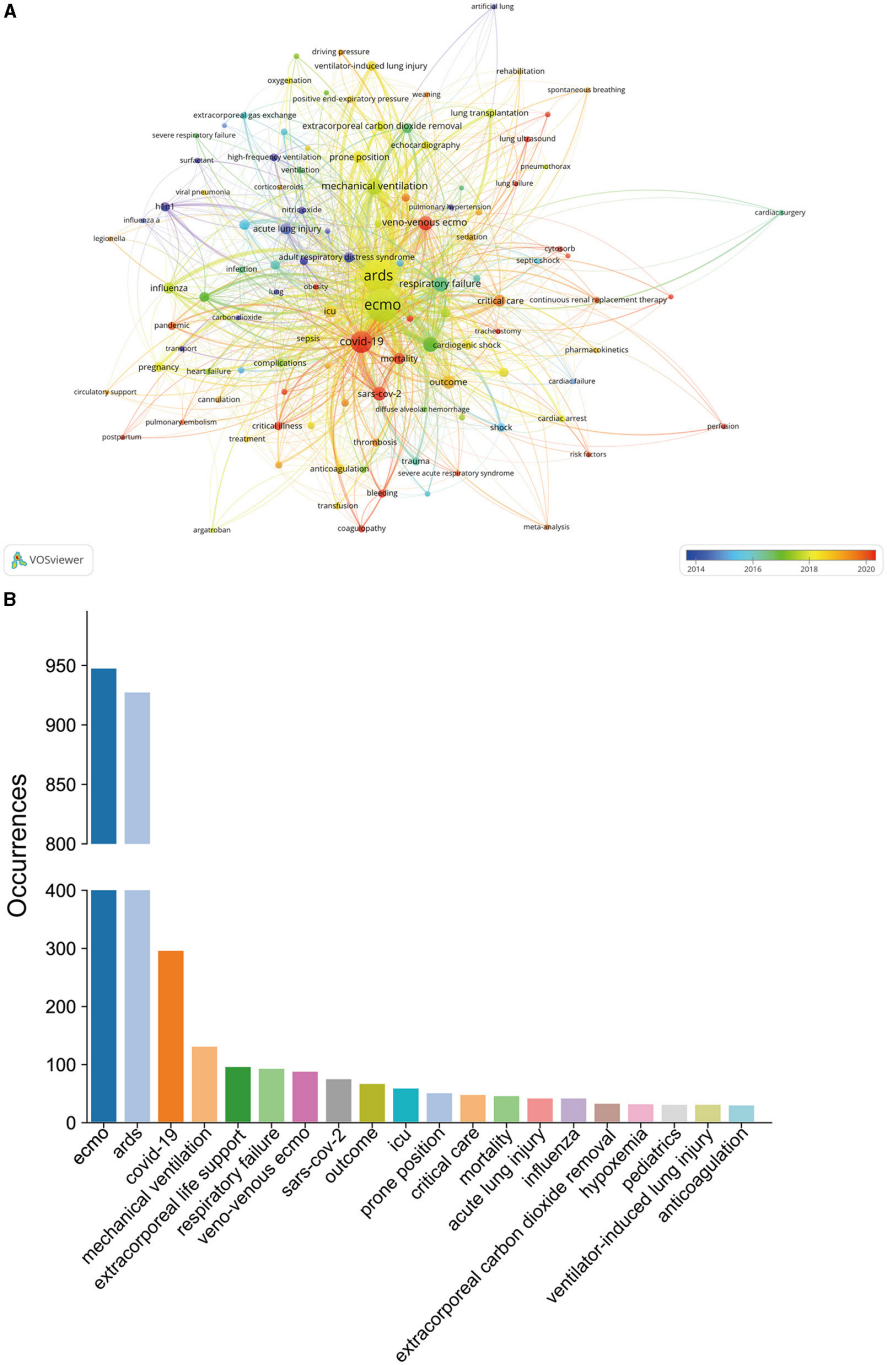
**(A)** Overlay visualization map of co-occurring keywords generated by VOSviewer. All these keywords were marked with different colors based on their AAY (the color gradient). Keywords that appeared relatively earlier were marked in blue, whereas keywords with a more recent appearance were marked in red. **(B)** Frequency distribution of the top 20 most frequently occurring keywords.

**Figure 9:**
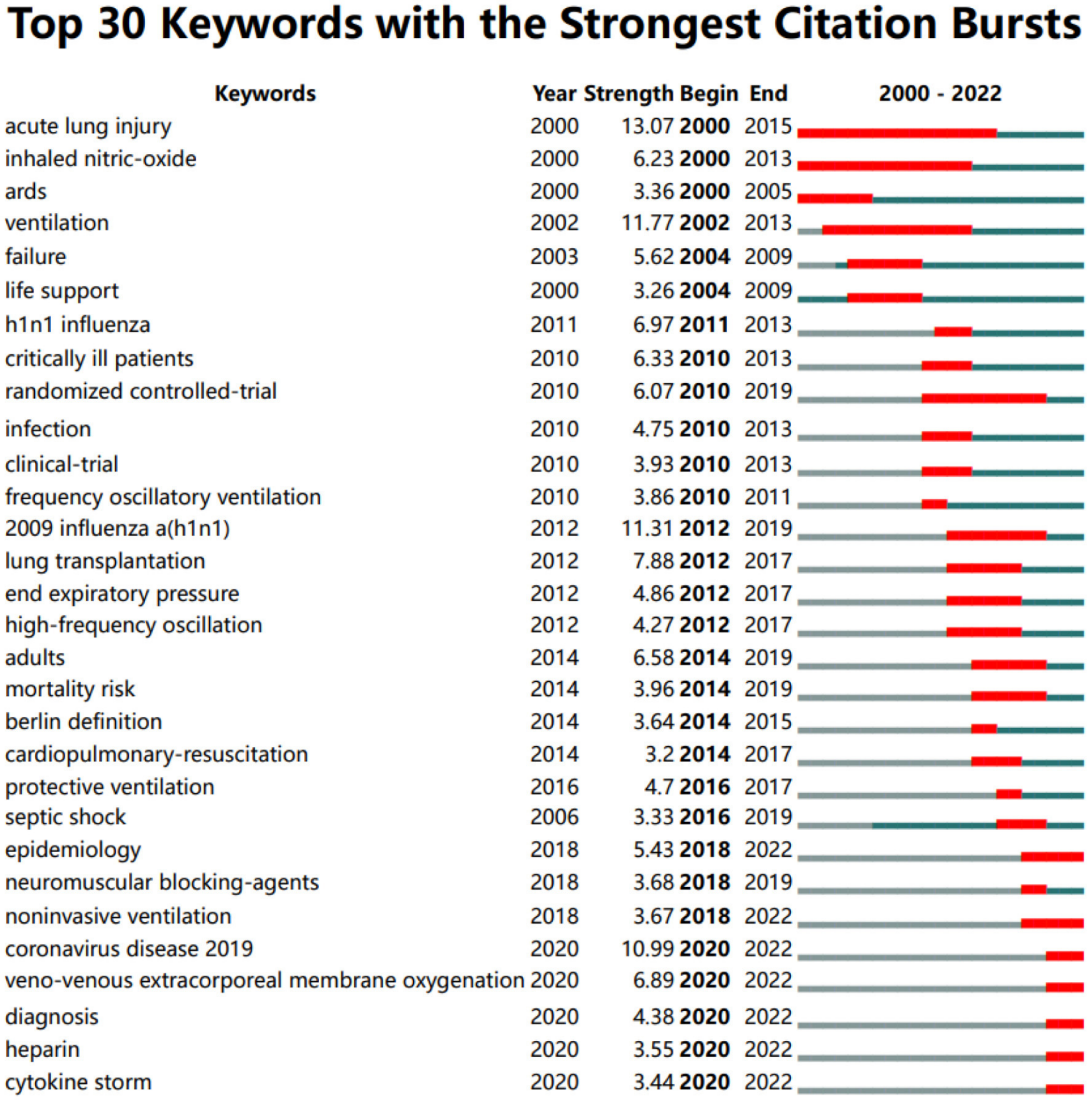
Top 30 keywords with the strongest citation bursts.

For this study, author keywords were extracted from all the 1,565 docunments. After excluding meaningless keywords and merging similar ones, a total of 115 meaningful keywords were identified. An overlay visualization map of these keywords was presented in Figure 8A. Additionally, Figure 8B illustrated the frequency distribution of the top 20 most studied keywords. As can be seen that, apart from “ECMO” and “ARDS”, the other common keywords in the research field were “COVID-19”, “mechanical ventilation”, “extracorporeal life support”, “respiratory failure”, “veno-venous ECMO”, “SARS-CoV-2”, “outcome”, and so on. In Figure 9, we presented the top 30 keywords with the strongest citation bursts spanning from 2000 to 2022. Remarkably, it is interesting to note that the citation burst time for several keywords has continued up to 2020.

## Discussion

Globally, the research papers on ECMO-ARDS have been increasing from 2 in 2000 to 270 in 2022, with particularly large increases during the last 3 years. These results reflect the continuous interest and recognition of ECMO therapy for ARDS management, making it a crucial and active area of investigation in the medical field. In addition, based on the annual number of documents, we roughly divided the whole period into three stages. The first stage was from 2000 to 2009. This initial stage represents the early years of research on ECMO-ARDS. During this period, the number of publications and citations might have been relatively low as the field was still developing and gaining attention. ECMO devices were developed in the 1960s and first performed by Gibbon (33) during cardiac surgery. In order to facilitate global application of ECMO technology, the International Extracorporeal Life Support Organization (ELSO), an international consortium of health care institutions dedicated to improve care and outcomes for patients receiving extracorporeal life support or ECMO, was established in 1989 spearheaded by Professor Robert H. Bartlett. However, even it reached the year 2000, the majority of studies on ECMO conducted earlier primarily from western developed countries. In April 2009, the Mexican Ministry of Health reported a surge in severe pneumonia cases among young adults. The cause was identified as a novel swine-origin influenza A (H1N1) virus, which rapidly escalated into a global pandemic (34). Subsequently, this virus spread to Australia and New Zealand during the southern hemisphere winter, and was also associated with a significant increase in the number of patients admitted to intensive care units (ICU) in both countries. After the successful use of ECMO in rescuing ARDS patients with H1N1 influenza in 2009, there has been a rapid increase in the global number of ECMO case around the world (35–37).

From 2010 to 2019, the utilization of ECMO in the treatment of ARDS experienced a consistent and notable increase, with the number of published articles rising from 37 in 2010 to 105 in 2019. This steady growth can be attributed to the progressive refinement and implementation of ECMO clinical trials and the widespread adoption of ECMO technology in intensive care units worldwide. For instance, a landmark international multicenter randomized controlled trial (RCT) conducted in 2018, known as the EOLIA trial (NCT01470703), included 249 patients (125 in the conventional treatment group and 124 in the ECMO group) (11). The trial results indicated that ECMO did not significantly improve the 60-day survival rate in adults with severe ARDS (46% vs. 35%, *P* = 0.09). Although the study did not meet its primary endpoint of a 20% reduction in hospital mortality for severe ARDS patients treated with ECMO, 28% of patients in the conventional treatment group received rescue ECMO therapy. The publication of these findings sparked considerable debate within the academic community (38, 39). However, subsequent efficacy and meta-analysis studies have demonstrated that ECMO can indeed save the lives of some patients with severe ARDS (12). Therefore, the prevailing view among scholars remains that traditional treatments for severe ARDS in adults are often inadequate, and ECMO continues to be regarded as a viable salvage and rescue therapy option.

The third stage was the rapid development period from 2020 to 2022. The third stage marks a period of significant growth and development in ECMO-ARDS research. The number of publications and citations likely increased substantially, indicating a growing interest and recognition of the field within the scientific community. Undoubtedly, the outbreak of the COVID-19 at the end of 2019 brought ECMO into the public eye as it was employed in the management of critically ill COVID-19 patients. Its crucial role in the treatment of ARDS patients during the pandemic highlighted the significance and potential of ECMO therapy in combating severe respiratory conditions (6, 7). Multinational clinical guidelines have recognized ECMO as a vital treatment technology for critically ill patients with COVID-19, leading to a significant increase in the establishment of ECMO centers in medical institutions worldwide (40, 41). Additionally, this recognition has also spurred the implementation of ECMO-related training and educational programs to enhance the expertise and proficiency of healthcare professionals in managing ECMO therapy effectively (42). Since then, the field of ECMO-ARDS has entered a rapid development period. The number of publications and citations likely experienced exponential growth.

Funding support is one of the main drivers of scientific research (43–46). Scientific research often requires funding to support various activities such as experiments, collecting data, and purchasing equipment and materials. Considering this, we have briefly summarized the top 6 related funding agencies in this field. Remarkably, 3 out of these agencies are headquartered in the United States. Therefore, this result may suggest that the leading position of the United States is inseparable from these funding support. In addition, previous studies showed that the discrepancy in the quantity of publications across different countries could be influenced by various factors, and one significant factor was the economy (47). When the publication productivity of worldwide countries/regions was compared, of the 15 most productive nations on ECMO-ARDS, apart from China, all other 14 were seen to be developed countries. Meanwhile, despite China being developing country, it was also country with large economy. This fact further suggests that the size of the economy has a significant impact on the global article productivity on the subject of ECMO-ARDS. Around the year 2000, ECMO technology in Europe and the United States had reached a level of maturity (48, 49). However, it resulted in the establishment of technical barriers and stringent restrictions on other countries concerning design, manufacturing, and core raw materials. One example of this is the dominant control by the US 3M company over the main material used in the core component membrane lung oxygenator. This long-standing monopoly of core materials, comprising more than 70% of the equipment production cost, has had a significant impact on the accessibility and affordability of ECMO equipment for other countries.

Highly cited papers are essential and valuable assets for bibliometric analysis. These papers are considered key indicators of research influence and impact within a specific field. Figure 7B showed the details of the top 15 highly cited studies in the research scope of ECMO-ARDS. Among these studies, the study authored by Wang D and colleagues, published in *JAMA*, holds the highest number of citations (30). In this retrospective, single-center case series of 138 hospitalized patients with confirmed COVID infected pneumonia in Wuhan, 36 patients (26.1%) were transferred to the ICU due to severe complications. Of them, ARDS comprised the largest portion of complications at 61.1% (22 cases). And 4 patients (11.1%) received ECMO as rescue therapy. The second-most cited study has evaluated the thrombotic risk in COVID-19 ARDS patients (31). Among 150 COVID-19 ARDS patients, 3 thrombotic occlusions of centrifugal pump occurred in 12 patients (8%) supported by ECMO. Additionally, the third and tenth ranked articles further investigated the role of ECMO support in COVID-19 patients (32, 50). An important conclusion drawn here is that in the subset of patients with COVID-19 receiving VV-ECMO and characterized as having ARDS, the estimated cumulative incidence of in-hospital mortality 90 days after the initiation of ECMO was 38.0%. In a meta-analysis, their findings showed that when compared with conventional mechanical ventilation, the utilization of VV-ECMO in adults with severe ARDS was linked to a reduction in 60-day mortality (12). All these results provide credible support for the existence of a survival benefit of ECMO in treating refractory ARDS.

In addition, there were also 3 studies reported the characteristics of patients with 2009 influenza A(H1N1)-associated ARDS treated with ECMO and their incidence, resource utilization, mortality and patient outcomes (51–53). These results further illustrated that the emergence of both H1N1 and COVID-19 has brought tremendous opportunities for the development ECMO, and we divided the whole research period into three stages was rational. The large-scale epidemic of H1N1 and COVID-19 has led to an increase in the demand for ECMO equipment and technology, which has promoted the continuous development and improvement of ECMO technology. Medical institutions and research institutions have increased their investment in ECMO and promoted scientific research and clinical practice in related fields. Meanwhile, the outbreak of these diseases has also accelerated the formulation of norms and guidelines for ECMO treatment in the medical community, improving the quality and safety of ECMO treatment. In addition, the experiences of fighting both epidemics remind that, during the outbreak of emerging infectious diseases, providing complex therapies such as ECMO faces unique challenges. Thorough planning, judicious allocation of resources, and personnel training are essential components of an ECMO action plan to deliver sophisticated therapeutic interventions while adhering to strict infection control measures.

Reference co-citation analysis is a valuable method for assessing advancements and identifying focal areas within a particular field. The timeline view of co-cited references provides a visual representation of the evolutionary trajectory of each cluster. As can be seen from Figure 7C, before 2010, the topics appearing more often were “high survival” (#9), “artificial lung” (#0), and “nonventilatory” (#10). After 2018, the more common subjects were “COVD ARDS” (#3), “COVID-19 patients” (#5), “multi-centered prospective” (#7), and “prone positioning” (#8) implying that the issues of the above clusters were the current research focuses in this field. Of them, prone positioning is considered a beneficial therapeutic measure that could promote alveolar recruitment and improve patient outcomes during ECMO support (54, 55). ECMO technology provides the lungs with an opportunity to rest, allowing patients to recover from life-threatening hypoxemia. The combined use of prone positioning with ECMO has been widely recognized and proven to be a safe and effective treatment modality, facilitating improved gas exchange and enhancing patient oxygenation and ventilation status. According to multiple meta-analysis, the application of prone positioning in ARDS patients during VV-ECMO was associated with better survival rates (56). Meanwhile, a longer duration of prone positioning with more than 12 h might improve the outcome of patients with ARDS who received VV-ECMO therapy (57). These finding further supported the positive role of prone positioning in ECMO therapy for ARDS.

Moreover, references with the strongest citation bursts first appeared in 2010, attributed to an article published in 2009. Also, this study published by Peek et al. (58) in Lancet had the highest burst value of 67.51. This study has compared the safety, clinical efficacy, and cost-effectiveness of ECMO with conventional ventilation support. The authors recommend transferring adult patients with severe but potentially reversible respiratory failure, whose Murray score exceeds 3.0 or who have a pH of < 7.20 on optimum conventional management, to a center with an ECMO-based management protocol. This approach has shown to significantly improve survival outcomes without severe disability. In addition, when a burst is still ongoing, it suggests that the research findings or ideas presented in those references continue to be influential and actively cited by other researchers. Therefore, although the burst in the majority of references has subsided, there are several references such as the studies by Combes et al. and Zhou et al., that continue to experience an ongoing burst, indicating that these research topics remain of continuous concern in recent years. Among them, the study conducted by Combes et al. (11) was an international clinical trial of ECMO for very severe ARDS, known as (EOLIA) trial. The results showed that the 60-day mortality rate was 35% in the ECMO group compared to 46% in the conventional management group, and this difference was statistically significant (relative risk, 0.76; *p* = 0.09). However, early application of ECMO was not associated with mortality at 60 days. Therefore, future studies are needed in the future to further investigate this subject.

In bibliometrics, another prevalent method for identifying hot research topics is through keyword co-occurrence analysis. In this type of analysis, the relatedness of keywords is determined based on the number of documents in which they appear together. In this study, we conducted a comprehensive keyword co-occurrence analysis to reveal prominent research directions and key areas in the field of ECMO-ARDS. As shown in Figure 8A, the size of each node in the map corresponds to the occurrence frequency of the respective keyword, while the relative distance between nodes indicates the strength of their relationship. According to the summary of the top 20 most common keywords from Figure 8B, As can be seen that, apart from the keywords “ECMO” and “ARDS” the other common keywords in the research field were “COVID-19”, “mechanical ventilation”, “extracorporeal life support”, “respiratory failure”, “veno-venous ECMO”, “SARS-CoV-2”, “outcome”, and so on. Generally, high-frequency keywords often indicate a hot topic in a particular field. Once again, this result demonstrated the important implications COVID-19 for the field of ECMO-ARDS. On May 5, 2023, the WHO has announced the COVID-19 epidemic would no longer be listed as a public health emergency of international concern, which considered as a symbol of the end of the global COVID-19 pandemic (59). However, reports on the new strain of the COVID-19 virus have never stopped (60). Concerns have been raised by several medical scholars regarding another outbreak. Therefore, we believe that ICUs department should strategically plan resource allocation to ensure that critical treatments like ECMO could be maximally utilized to face future challenges. Moreover, there are two main types of ECMO: Venoarterial (V-A) ECMO, which provides support for both the heart and lungs, and VV-ECMO, which is specifically used for lung support. Compared to V-A ECMO, VV-ECMO offers several distinct advantages, including reducing cardiac burden, providing pure respiratory support, and minimizing the risk of complications. Generally, VV-ECMO is more widely used, especially in cases of respiratory failure or ARDS. Compared to VV-ECMO, V-A ECMO has a narrower application range because its primary goal is to provide support for cardiac function, rather than pure respiratory support (7, 8). As this study primarily focuses on the analysis of ECMO treatment for ARDS, it is reasonable to find VV-ECMO as a high-frequency studied keyword.

Moreover, as can be seen in Figure 8A, all those keywords were also colored differently according to their AAY. A blue color indicates keywords appearing relatively earlier, while a red color indicates keywords appearing recently. During the early stage of research, keywords such as “H1N1”, “acute lung injury”, “high-frequency ventilation”, and “influenza”, et al., were prominent topics. On the other hand, keywords like “COVID-19”, “SARS-CoV-2”, “mortality”, “veno-venous ECMO”, “epidemiology”, “obesity”, “coagulopathy”, “lung ultrasound”, “inhalation injury” were marked with a relatively recent AAY. This indicates that these topics have gained increasing attention in more recent years, suggesting they have the potential to become research hotspots in the near future. Burst keywords is considered another important indicator of research frontiers and can provide insights into emerging tendencies in a certain field. According to the top 30 keywords with the strongest citation bursts in Figure 9. it is interesting to note that the citation burst time for keywords such as “epidemiology”, “noninvasive ventilation”, “coronavirus disease 2019”, “veno-venous extracorporeal membrane oxygenation”, “diagnosis”, “heparin”, “cytokine storm” has continued up to 2020, and the bursts are still ongoing, suggesting that these topics are currently active and attracting significant attention from researchers. Taken together, keywords like “mortality”, “veno-venous ECMO”, “epidemiology”, “obesity”, “coagulopathy”, “lung ultrasound”, “inhalation injury”, “noninvasive ventilation”, “diagnosis”, “heparin”, “cytokine storm” has received growing interest in current research and also has the potential to continue to become research hotspots in the near future. Meanwhile, over the past two decades, ECMO has undergone significant advancements, particularly in ECMO devices, such as centrifugal pump techniques, complete heparin-coated circuits, more efficient oxygenators, and device miniaturization. Despite the increasing utilization and evolving technology of ECMO, there remains a limited number of epidemiologic reports that comprehensively describe its uses and outcomes. More research and data collection are needed to gain a better understanding of the impact and effectiveness of ECMO in various clinical settings including ARDS.

## Limitations

This bibliometric analysis has provided valuable and objective insights into the evolving research hotspots and trends related to ECOM-ARDS. Similar to other bibliometric studies, our research also has some limitations. Firstly, we only searched the WoSCC database and selected articles written in English, which could lead to the exclusion of high-quality articles in other languages. Secondly, due to limitations in CiteSpace and VOSviewer software, the abbreviation of author names cannot be distinguished, which might introduce some bias in the analysis. Thirdly, the bibliometric method based on natural language processing is susceptible to biases stemming from subject categorization and citation behavior, which could influence the results to some extent. Nonetheless, the use of a sufficient sample size could help mitigate these biases and enhance the accuracy of the research findings to a certain degree. In addition, identifying remotely related papers, especially the COVID-19 pandemic with remotely related papers, may confound the findings and conclusions of the study, and even dilute the interesting research question of the paper, and a more robust approach in the future to identify relevant articles may improve validity.

## Conclusion

ECMO provides adequate oxygenation and carbon dioxide removal, offering a crucial life support option for critically ill ARDS patients. Over the past few decades, ECMO treatment for ARDS has seen remarkable advancements and continuous progress. Particularly during the COVID-19 pandemic, ECMO has played a pivotal role in the management of severe ARDS cases. Overall, to the best of our knowledge, this is the first study to demonstrate the global research trends and hotspots of ECMO-ARDS through a bibliometric analysis. Our results showed that the annual publication number in this field revealed exponential growth trends. In addition, we also summarized the main contributors, including authors, institutions, and countries in the field of ECMO-ARDS, the active journals that were preferred for publishing related documents, as well as the main research directions and hotspots currently and in the future. All in all, the research on ECMO therapy for ARDS has made remarkable progress to date with promising future trends. this bibliometric analysis offers a comprehensive understanding of the current state of ECMO-ARDS research and can serve as a valuable resource for researchers, policymakers, and stakeholders in exploring future research directions and fostering collaborations in this critical field.

## Data availability statement

The original contributions presented in the study are included in the article/supplementary material, further inquiries can be directed to the corresponding authors.

## Author contributions

KC: Methodology, Supervision, Validation, Visualization, Writing – review & editing. YL: Conceptualization, Data curation, Investigation, Methodology, Software, Validation, Writing – original draft. WL: Data curation, Methodology, Resources, Supervision, Validation, Writing – original draft. SQ: Conceptualization, Data curation, Formal analysis, Methodology, Supervision, Writing – review & editing. HW: Conceptualization, Formal analysis, Methodology, Supervision, Writing – review & editing.
